# Effectiveness of Acidic Chitosan Solutions for Total Organic Carbon Removal in Drinking Water Treatment

**DOI:** 10.3390/polym17131832

**Published:** 2025-06-30

**Authors:** Josefine Molina-Pinna, Félix R. Román-Velázquez

**Affiliations:** Department of Chemistry, University of Puerto Rico, Mayaguez Campus, Mayaguez, PR 00681, USA; felixr.roman@upr.edu

**Keywords:** chitosan in different acidic solutions, water treatment plant, high raw water turbidity, TOC, TOC removal, DBPs, sodium hypochlorite

## Abstract

Natural organic matter (NOM) in surface waters is a major challenge for drinking water treatment due to its role in the formation of disinfection byproducts (DBPs) during chlorination. This study evaluated the performance of chitosan, a biodegradable coagulant, dissolved in acetic, lactic, and L-ascorbic acids for NOM removal under three turbidity levels (403, 1220, and 5038 NTU). Jar tests were conducted using raw water from the Río Grande de Añasco (Puerto Rico), and TOC, DOC, and UV_254_ were measured at multiple time points. TOC removal ranged from 39.8% to 74.3%, with the highest performance observed in high-turbidity water treated with chitosan–L-ascorbic acid. DOC and UV_254_ reductions followed similar trends, with maximum removals of 76.4% and 76.2%, respectively. Estimated THM formation potential (THMFP) was reduced by up to 81.6%. Across all acids, flocculation efficiencies exceeded 95%. Compared to conventional aluminum-based coagulants, chitosan demonstrated comparable performance, while offering environmental benefits. These results confirm the potential of chitosan–acid systems for effective organic matter removal and DBP control, supporting their application as sustainable alternatives in drinking water treatment.

## 1. Introduction

**Figure 1:**
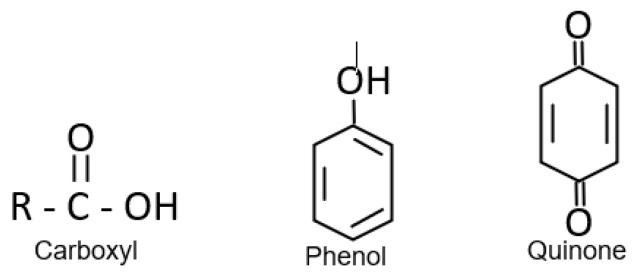
The functional groups that are part of humic substances.

Humic substances are a large part of the natural organic matter (NOM) compounds, produced by soil degradation, which in turn incorporates them into the water through runoff. Humic substances are the major component of dissolved organic carbon; they are part of the dead constituents of the soil, reaching values of 60 to 90 percent of the total carbon [1]. The chemical composition of humic substances varies depending on the soil. Humic substances are high-molecular-weight macromolecules, and their charge is mainly caused by carboxyl and phenolic groups and they contain carbohydrate and protein residues [2]. The functional groups that are part of humic substances, as shown in Figure 1, can be ionized, donating their H+ atoms and favoring an acidic environment. The general structures that occur for these substances are phenolic groups, quinones, nitrogen, and oxygen as bridges, and carboxylic acid groups in the aromatic rings. The functional groups that can be found are acetic acid, benzoic acid, phenol, and ethanol, among others.

NOM is composed of humic and non-humic substances. Humic substances such as fulvic and humic acids have hydrophobic characteristics. Non-humic substances include proteins, amino acids, and carbohydrates with less hydrophobic characteristics. The amount of NOM in water was calculated using the amount of organic carbon. TOC is the total concentration of organic carbon in water and represents a large part of the NOM constituents; thus, TOC measurement is used as an optimization measure in drinking water treatment systems to predict the formation of disinfection byproducts [3]. The average concentration of total organic carbon (TOC) in surface water is 4 mg/L [4]. The organic compounds in NOM are aromatic, with conjugated double bonds that absorb light in the visible ultraviolet range, typically at 250 nm. Waters with low concentrations of humic acids generally also have low concentrations of TOC (<2 mg/L).

Chlorine is primarily used for its ability to inactivate pathogens, allowing residual chlorine levels in distribution water. The amount of DBPs formed by the reaction with disinfectant chemicals, such as chlorine, is proportional to the amount of organic carbon in the water [5].

**Figure 2:**
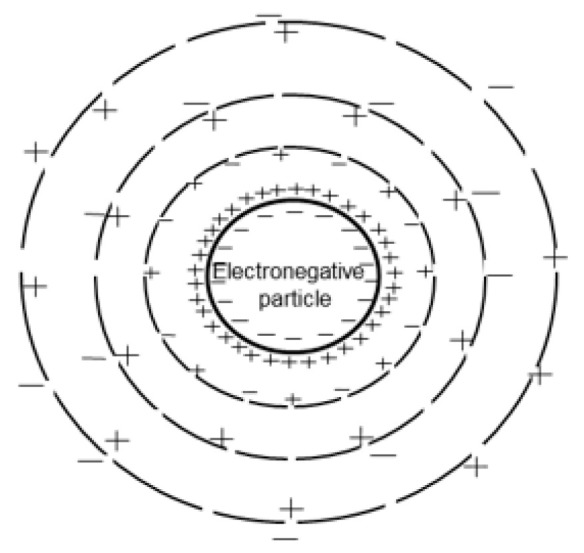
Charge attraction between the coagulant and the humic acids.

The drinking water treatment process includes the stages of coagulation, which involve the destabilization of the particle and enhance the rate of floc formation. In this process, the particles in a stable suspension are modified to increase their tendency to attach to one another [5]. The attraction of the charges between the coagulant and humic acids is shown in Figure 2.

**Figure 3:**
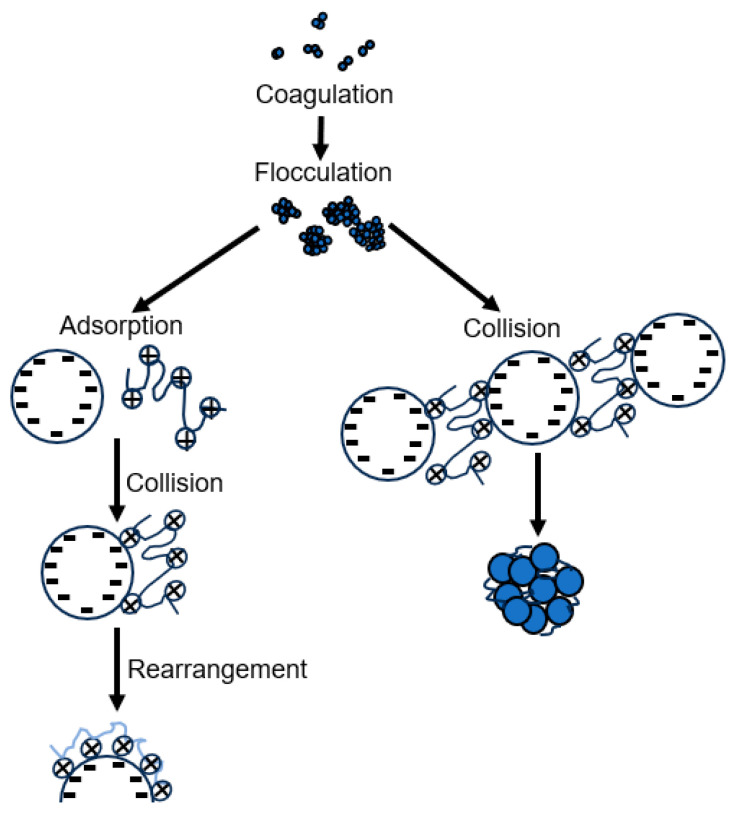
Bridging and neutralization mechanisms.

The presence of large polymer chains in water produces larger and stronger flocs. When the flocs are large, spherical in shape, and have a packed structure, they are called floc pellets. The flocculation process involves physicochemical conditions and mechanisms for its formation, which are called bridging and neutralization. Flocculation can be achieved depending on the predominant mechanism. When the added polymer chain is long and has a high molecular weight, loops are formed, which can reach the surface of the particles. The unoccupied spaces on the surface of the particle are where the polymer chain is adsorbed, and the union of particles can occur, forming large flocs and giving rise to the bridging process. The neutralization process is based on the adsorption of a highly charged polymer by the oppositely charged surface of the particle, thereby reducing the net charge of the particle. This neutralization of the particle charge reduces electrostatic repulsion, preventing the particles from repelling each other and allowing the formation of large flocs. Both mechanisms are based on polymer adsorption and particle collision. A representation of these mechanisms is shown in Figure 3.

Turbidity is a key indicator of water quality, representing the cloudiness or haziness of a fluid caused by suspended particles that scatter and absorb light rather than allowing it to pass through in a straight line. These particles may include clay, silt, organic matter, microorganisms, and other finely divided solids.

Turbidity is not a direct measure of the concentration of particles, but rather of the optical property of the water—that is, the intensity of light scattered at a 90-degree angle by particles in the water when a light source passes through it. The standard unit used to quantify turbidity is the nephelometric turbidity unit (NTU), which is derived from a nephelometric technique that measures the intensity of light scattered at a fixed angle, typically 90°, from an incident light source using a nephelometer or turbidimeter. Turbidity values are commonly interpreted according to their clarity levels: values between 0 and 1 NTU correspond to very clear drinking water; values from 1 to 5 NTU indicate slightly turbid water, which is generally acceptable for most uses but requires treatment prior to consumption; values above 5 NTU reflect increasingly cloudy conditions that may suggest potential contamination or elevated sediment loads; and values exceeding 1000 NTU are classified as extremely turbid, typically observed in surface waters during high-flow events or after heavy rainfall, and usually demanding intensive treatment efforts. High turbidity can interfere with disinfection processes and may harbor pathogens by providing physical protection. It also affects the aesthetic quality of the water and can indicate upstream erosion, pollution, or runoff events.

In this study, turbidity was measured using a calibrated nephelometer and values were recorded at different stages of the treatment process to assess the effectiveness of the flocculant product under varying raw water conditions. Although the focus of this study was the removal of total organic carbon (TOC), turbidity measurements throughout the treatment process provide a complementary tool for evaluating the efficiency of the flocculant. This is because natural organic matter, which represents a significant portion of the TOC, is often associated with colloidal or suspended particles that contribute to water turbidity. Therefore, a reduction in turbidity may correlate with the removal of organic compounds, particularly during the initial stages of treatment, such as coagulation and flocculation. For efficient flocculation, the particle suspension must be destabilized by adding flocculant as a synthetic organic compound. The efficiency of the flocculation product (EFP) was calculated by measuring the inlet (NTU RW) and outlet turbidity of the flocculation unit (NTU out) at intervals of 30 min, which is the standard time for the flocculation process [5].

Polymers with high molecular weight and synthetic organic compounds that have a strong tendency to be adsorbed on the surface of the particles in an aqueous suspension are used for the flocculation process. A flocculant is used after the addition of a coagulant to enhance floc formation and increase the strength of the floc structure [5]. The purpose of flocculation is to obtain particles that interact with each other and form larger floccules that can be efficiently removed by separation processes such as sedimentation [5].

Inorganic coagulants based on aluminum and ferric compounds are the most widely used coagulants. The study of alternative coagulants to aluminum-based compounds has gained relevance owing to the drawbacks associated with their use. Among these issues are the high concentrations of heavy metals that reach water bodies receiving effluents from treatment plants and the large doses required to achieve effective coagulation.

Therefore, chitosan has emerged as a promising alternative. Various studies have shown that this biopolymer, derived from chitin, demonstrates effectiveness similar to that of aluminum-based polymers as well as iron-based chemical coagulants in terms of its ability to remove turbidity and organic load from water [6]. Chitosan is the second most common natural polymer after cellulose [7]. It is a non-toxic, linear, high-molecular-weight cationic polymer. Chitosan is insoluble in water, alkalis, dilute acids, alcohols, and other organic solvents. When using organic or inorganic acids, the free amino groups are protonated, and the biopolymer becomes fully soluble. Chitosan has the advantage of being a biodegradable material, making it a more environmentally friendly option than synthetic coagulants. This natural coagulant not only provides efficient treatment, but also minimizes long-term ecological impacts, opening new possibilities for improving water purification processes.

Several studies have evaluated the use of chitosan as a coagulant in water treatment applications; however, none have specifically focused on total organic carbon (TOC) removal. In 2014, Bina et al. demonstrated that optimized doses of chitosan and ferric chloride achieved turbidity removal efficiencies of up to 95% in jar tests using raw water with turbidity between 20 and 200 NTU [8], although their study did not include TOC removal as a key parameter. Similarly, a 2012 study by Mega Ng et al. investigated a composite coagulant consisting of polyaluminum chloride (PACl) and chitosan, showing improved removal of natural organic matter (NOM) from synthetic water based on UV254 absorbance and dissolved organic carbon (DOC) measurements [9].

More recently, in 2023, a study conducted in Japan evaluated chitosan as a coagulant aid alongside PACl and concluded that chitosan could achieve comparable treatment performance with reduced health risks. Although this study indicated the effective removal of particulates, it did not specifically address TOC removal [10]. Furthermore, in 2016, a study by Abebe et al. at the University of North Carolina assessed chitosan in household water filtration systems. Chitosan significantly enhanced the removal of Escherichia coli and viruses, in addition to reducing turbidity; however, the study did not investigate TOC removal, focusing primarily on microbial and turbidity reduction [11]. These studies highlight the effectiveness of chitosan in various water treatment applications, yet they do not provide specific data on its potential for TOC removal, which is a key focus of the current research. Despite these promising results, few studies have focused specifically on the effectiveness of chitosan in the removal of TOC, particularly from raw surface water sources with three different turbidity ranges. This gap is significant, given the role of TOC as a key precursor to DBPs. Therefore, evaluating the performance of chitosan in reducing TOC may offer a more complete understanding of its potential as a natural coagulant. Water samples were collected from the Río Grande de Añasco at the intake point of the municipal water treatment plant (MWTP) in Puerto Rico. Jar tests were conducted using chitosan solutions prepared with different organic acids, including acetic acid, lactic acid, and l-ascorbic acid. TOC concentrations were measured at three critical stages of the treatment process: raw water, flocculation, sedimentation, and filtration, using a 1:50 scale model filter.

## 2. Materials and Methods

### 2.1. Materials

Commercial chitosan powder (C3646) obtained from shrimp shells was acquired from Sigma-Aldrich (St Louis, MO, USA), with a degree of deacetylation (DD) ≥ 75% and molecular weight of 460 KDa [12]. L-ascorbic acid (99%, 255,564), acetic acid (99.7%, 64-19-7), and lactic acid (86%, 79-33-4), were acquired from Sigma-Aldrich (USA). Hydrochloric acid (37%, 7732-01-0) was acquired from Across Organics (Burlington, MA, USA). The turbidity meter used was TL2300 [13]. A Hach Pocket Colorimeter was used to analyze residual chlorine [14] and a HQ40d Hach Meter [15] was used to analyze the pH and temperature. The jar test unit, using 1 L jars, was from MCR Technologies [16]. The total organic carbon (TOC) analyzer used was Sievers M5310 C [17]. The UV_254_ P series, P200 model [18], was used for absorbance measurements. A 1:50 scale filter model with anthracite, sand, and gravel was used to simulate the filtration process during the jar tests.

### 2.2. Characterization of Natural Organic Matter (NOM) from the Añasco River

Specific ultraviolet absorbance (SUVA) was used as an indicator of the aromaticity and hydrophobic character of dissolved organic matter (DOM). SUVA is calculated by dividing UV absorbance at 254 nm (UV_254_) by the dissolved organic carbon (DOC):

SUVA = (UV_254_/DOC) × 100 (1)

where UV_254_ is in cm^−1^, DOC is in mg/L, and SUVA is expressed in L/mg·m.

### 2.3. Theoretical TOC Removal and Alkalinity as an Indicator

The theoretical percentage of TOC removal was estimated using the equation that relates specific ultraviolet absorbance (SUVA) to the TOC of the raw water (TOC RW) [19,20]:

Theoretical TOC removal (%) = 10.8 (SUVA)^1.32^ (2)

### 2.4. Potential Formation of Trihalomethanes (THMFP)

Based on previous studies on surface waters, a correlation was established between total organic carbon (TOC) and the formation of trihalomethanes (THMs), which are regulated disinfection byproducts (DBPs). Canale [21] and Letterman [5] described this relationship using an empirical equation derived from chlorination experiments under controlled conditions. This correlation allows for the estimation of trihalomethane formation potential (THMFP) using TOC concentrations. The general form of this relationship is expressed as follows:

THMFP = 43.78 × (TOC)^1.248^ (3)

where THMFP is the estimated trihalomethane formation potential in µg/L, and TOC represents the total organic carbon concentration, either in raw water (TOC_RW) or in finished water (TOC_FW), depending on the stage of evaluation. The constants and exponents used in the equation were derived under typical chlorination conditions (free chlorine dose ~3 mg/L, pH ≈ 7–8, temperature ≈ 20–25 °C). This equation was applied in this study to estimate THMFP values both before and after treatment with chitosan-based coagulants. While the model was originally developed using raw water TOC data, its application to finished water TOC has been supported in recent DBP risk assessment studies to evaluate the treatment’s effectiveness in reducing disinfection byproduct precursors.

### 2.5. Scale Filter Model

**Figure 4:**
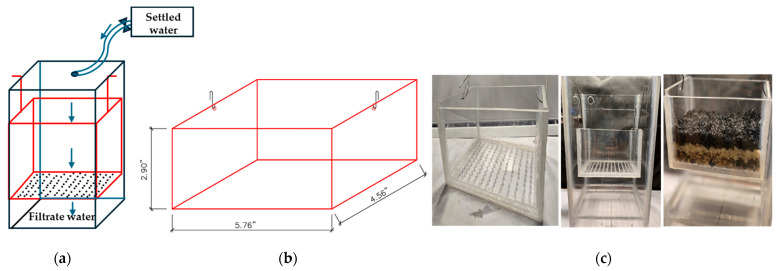
(**a**) Flow diagram (not to scale); (**b**) Mobile filtration unit (with dimensions); (**c**) Photograph of the constructed model used in the study.

A scale model of the filtration system was constructed at a 1:50 scale to simulate the hydraulic behavior of the full-scale filter. The model was designed to replicate the geometric dimensions and hydraulic characteristics of the original filter, ensuring the preservation of the critical flow dynamics and filtration processes. The media layers, including anthracite and sand, were kept to the original particle sizes to maintain hydraulic similarity, with the effective size of the sand being 0.45 mm and the anthracite 0.9 mm. The gravel support layer was adapted owing to size limitations in the model. Figure 4a,b show the flow diagram and the mobile filtration unit (with dimensions), respectively, while Figure 4c displays a photograph of the constructed model used in the study.

### 2.6. Chitosan Stock Solutions—Preparation Procedure

Four stock chitosan solutions (1000 mg/L) were prepared using different acids. First, acid solutions were prepared by dissolving 6 mL of acetic acid, 1 mL of lactic acid, and 0.5–1 g of L-ascorbic acid, respectively, in 900 mL of deionized water in 1000 mL volumetric flasks. The pH of each solution was adjusted to between 3.5 and 4.5 to ensure adequate dissolution of chitosan. Subsequently, 1 g of chitosan powder was slowly added to each acidic solution with stirring to prevent lump formation. The mixtures were magnetically stirred for 30 min to ensure complete dissolution. After stirring, the volume of each solution was brought to 1000 mL with deionized water. The prepared solutions were stored at room temperature for 48 h prior to use.

### 2.7. Total Organic Carbon Removal Using Chitosan in Different Acidic Solutions

#### 2.7.1. Collection and Preparation of Samples for Jar Test

Raw water samples were collected from the Río Grande de Añasco at the intake point where water enters the municipal water treatment plant (MWTP) and placed into 1000 mL jars. The following were added to each jar: varying doses of chitosan acid solution (mg/L) and a consistent dose of sodium hypochlorite (SH) (mg/L). The jar test procedure and dose selection logic included a two-stage jar test conducted to determine the optimal coagulant dosage. In the first stage, a wide range of dosages was tested to evaluate the response of the system. The second stage involved a more focused set of jar tests using doses close to the initially estimated optimum. This step aimed to refine the dose selection with greater accuracy and to confirm that the optimal dosage was not chosen arbitrarily or based on a single data point. Instead, the process demonstrates intentional optimization. The final coagulant dose selected for total organic carbon (TOC) analysis resulted in the lowest turbidity. This criterion is justified, as turbidity is generally correlated with particulate organic matter, which is a major contributor to TOC. Applying TOC analysis only to the most efficient dose is a practical and scientifically sound approach considering the high cost and time requirements of TOC measurements.

#### 2.7.2. Jar Test Analysis

The jar test process replicated the actual treatment method used at the MWTP and followed a structured mixing sequence. Rapid mixing was conducted for 1 min at 100 RPM to disperse the coagulant evenly, followed by a slow mixing phase of 32 min at 30 RPM to promote floc formation. This was followed by a sedimentation phase lasting 178 min, during which no stirring was applied (0 RPM) to allow the flocs to settle. After sedimentation, the supernatant was passed through a 1:50 scale filter to simulate the filtration process. Water samples were collected 15 min after filtration, totaling 193 min from the beginning of the process, and then subjected to further analysis.

#### 2.7.3. Sample Analysis

Samples were analyzed in triplicate both before the addition of chitosan and after the jar test. Residual chlorine was measured using the 4500-Cl(E) method, which is a USEPA-approved colorimetric method and standardized operational protocol [22]. TOC concentrations were determined using a Sievers M5310C TOC Analyzer [17]. For UV_254_ analysis, a UV_250_ field meter was employed; samples were filtered through a 0.45 µm membrane to eliminate colloidal interference prior to analysis. This method quantifies ultraviolet light absorbance at a wavelength of 253.7 nm (UVA) with a resolution of 0.001 and accuracy of ±0.5, using a low-pressure mercury UV lamp or UV LED as the light source [18]. pH and temperature were measured using the HQ40d Hach multiparameter meter [15], and turbidity was measured with a TL2300 turbidimeter [13].

#### 2.7.4. Efficiency of the Flocculation Process

To assess the performance of the flocculation product, turbidity readings were taken at the inlet (NTU RW) and outlet (NTU out) of the flocculation unit at 30 min intervals, which corresponds to the typical duration of a complete flocculation cycle, as described by Letterman [5]. These measurements were used to calculate flocculation efficiency under varying raw water conditions. The efficiency of flocculation (EFP) was calculated using the following equation:

EFP (%) = [(NTU RW − NTU out)/NTU RW] × 100(4)

#### 2.7.5. Experimental Percentage of TOC Removal (% TOC Removal Exp)

The experimental percentage of TOC removal (% TOC removal exp) was calculated from the data obtained using Equation (5):

% TOC removal exp = [(TOC RW − TOC after jar test)/TOC RW] × 100(5)

## 3. Results

### 3.1. Characterization of NOM in Río Grande de Añasco

Río Grande de Añasco, located in western Puerto Rico, is known for its high turbidity and sediment load, particularly during the rainy season from May to November. The Mayagüez Water Treatment Plant (MWTP) faces significant challenges in treating raw water due to the wide range of turbidity levels observed.

**Figure 5:**
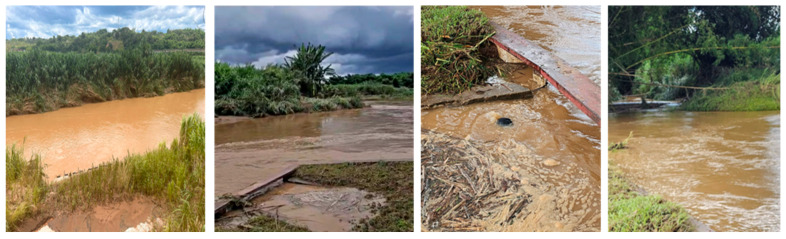
Appearance of the Río Grande de Añasco during a high-turbidity event (5038 NTU).

This study was conducted using raw water from Río Grande de Añasco under three different turbidity conditions: 403, 1220, and 5038 NTU. The turbidity levels were deliberately selected to represent different stages of intense and prolonged rainfall events forecasted by the national meteorological center for the Rio Grande de Añasco basin. These values correspond to early, mid, and peak turbidity conditions typically observed during such storm events. The rationale behind choosing these turbidity points is based on the challenge posed by the initial rainfall in a multi-day event. During the first rains, there is often a significant mobilization of organic matter and suspended particles from the watershed, which complicates coagulation and flocculation processes aimed at removing total organic carbon (TOC) and aromatic compounds. By evaluating treatment efficiency at these representative turbidity levels, the study provides insights into the performance of chitosan-based coagulants under realistic and critical water quality conditions that impact water treatment operations. Figure 5 shows photographs of the river during the event in which raw water turbidity reached 5038 NTU, illustrating the extreme sediment load present under such conditions.

Specific ultraviolet absorbance (SUVA) was calculated to assess the molecular weight characteristics of the natural organic matter (NOM) present in the raw water samples. As described by Edzwald [3], SUVA was obtained by dividing the UV absorbance at 254 nm (UV_254_) by the corresponding dissolved organic carbon (DOC) concentration, both measured in raw water, where UV_254_ is in m-1 and DOC in mg/L, using Equation (1).

Humic substances, in particular, are the major precursors of disinfection byproducts (DBPs). Higher SUVA values (>2.0 L/mg·m) indicate a greater presence of hydrophobic, aromatic NOM, which is more reactive with chlorine and is thus more likely to form DBPs. According to EPA guidelines [19], a decrease in SUVA after treatment is a strong indicator of effective precursor removal. Therefore, comparing SUVA values before and after coagulation provides insight into the removal of aromatic DOM and the potential reduction in THM formation. The typical ranges of SUVA can be used to characterize the type of NOM present and its expected removability through coagulation. SUVA is directly proportional to UV_254_ absorbance, which reflects the presence of conjugated double bonds in aromatic structures [20]. Table 1 summarizes the physicochemical parameters and specific ultraviolet absorbance (SUVA) values of the raw water samples selected for the jar test experiments. These values reflect the variability in the NOM characteristics under different turbidity conditions. SUVA values were calculated using Equation (1).

### 3.2. Theoretical Percentages of TOC Removal and Alkalinity as an Indicator

The theoretical percentage of TOC removal was estimated using Equation (2), which relates the specific ultraviolet absorbance (SUVA) to the TOC of raw water (TOC RW) [3]. The SUVA values used to calculate theoretical TOC removal are listed in Table 1. The specific ultraviolet absorbance (SUVA) values calculated for the raw water samples are presented in Table 2, along with the corresponding theoretical TOC removal percentages and expected removal ranges based on EPA guidance.

All three samples exhibited SUVA values greater than 2 L·mg^−1^·m^−1^, indicating the presence of predominantly hydrophobic and humic substances. Specifically, the samples with turbidities of 403 and 1220 NTU had SUVA values of 3.58 and 3.61 L·mg^−1^·m^−1^, respectively, placing them in the intermediate aromaticity range (40–60% expected TOC removal). The calculated theoretical removals of 58.1% and 58.8% were consistent with this classification. In contrast, the sample with the highest turbidity (5038 NTU) showed a SUVA of 4.39 L·mg^−1^·m^−1^, reflecting high aromatic content and dominance of humic, hydrophobic NOM. This sample corresponded to an expected TOC removal range of 60–80%, with a calculated theoretical removal of 76.1%.

These results support the interpretation that the nature of the organic matter shifts with turbidity and that higher SUVA values correlate with increased removal requirements and a greater proportion of humic substances. Furthermore, when considering the initial TOC concentrations (ranging from 3.67 to 8.00 mg/L) and raw water alkalinity (76–80 mg/L), the EPA-specified minimum TOC removals of 25–35% were clearly exceeded in theoretical terms. This alignment underscores the suitability of enhanced coagulation strategies for improving these water qualities. These thresholds serve as benchmarks for assessing the effectiveness of the coagulation treatments applied in this study and provide a context for comparing the actual TOC removal results.

### 3.3. Trihalomethane Formation Potential (THMFP) as a Function of Turbidity and TOC Removal

Based on previous studies on surface waters, a correlation was established between the TOC RW and the formation of trihalomethanes (THM) [21]. The relationship is given by Equation (3) for the trihalomethane formation potential [5]. The relationships between raw water turbidity, theoretical TOC removal, and trihalomethane (THM) formation potential are summarized in Table 3. As turbidity increased from 403 to 5038 NTU, both the theoretical TOC removal percentage and THM formation potential also increased significantly. Specifically, the sample with the highest turbidity (5038 NTU) showed a theoretical TOC removal of 76.1% and a THM formation potential of 587 µg/L, compared with 58.1% and 222 µg/L in the sample with 403 NTU. This trend reflects the greater presence of reactive organic matter, particularly aromatic and humic substances, in highly turbid waters, as indicated by the SUVA values.

These results highlight the dual challenge posed by elevated turbidity: not only is a higher removal efficiency required to meet regulatory standards, but the associated risk of DBP formation is also markedly increased. Therefore, optimizing coagulation and organic matter removal is essential for reducing THM precursors, especially in waters with elevated particulate and humic contents.

### 3.4. The Experimental Percentage of TOC Removal Using Chitosan in Different Acidic Solutions

To prepare stock chitosan solutions (1000 mg/L), four acid solutions were formulated using acetic, lactic, and l-ascorbic acids. Depending on their physical state and purity, each acid was either weighed or measured by volume to achieve a target pH range of approximately 3.5–4.5. The acid was first dissolved in 900 mL of deionized water and stirred for 30 min using a magnetic stirrer. Approximately 1 g of chitosan was then added to each solution. The final volume was adjusted to 1000 mL in a volumetric flask and the solutions were shaken using a magnetic stirrer for 20 h. As shown in Table 4, the pH values of all solutions reached an average of approximately 3.70, which was within the optimal range for effective chitosan solubilization.

#### 3.4.1. Collection and Preparation of Samples for Jar Test

Blank jar control setup and monitoring

**Figure 6:**
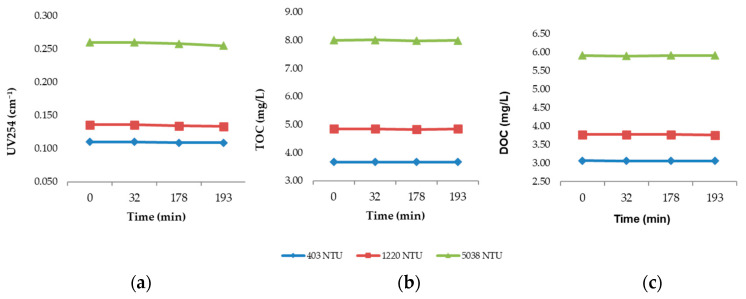
Variation of (**a**) UV_254_ absorbance, (**b**) total organic carbon (TOC), and (**c**) dissolved organic carbon (DOC) in blank jars containing untreated raw water at three turbidity levels (403, 1220, and 5038 NTU) over 193 min.

Raw water samples were collected from the Río Grande de Añasco at the intake point of the municipal water treatment plant (MWTP) and transferred into 1000 mL jars. A blank jar was prepared using untreated raw water at three turbidity levels (403, 1220, and 5038 NTU) and monitored over a period of 193 min. This duration was selected to simulate the contact and sedimentation time used in the jar tests with coagulant addition (chitosan in different acidic solutions and aluminum chlorohydrate as the conventional comparator). The temporal evolution of UV254, TOC, and DOC in the blank jars is presented in Figure 6a, b, and c, respectively.

As expected, the results indicate only minimal variation in all three parameters over the 193 min period, confirming that the observed removals in treated samples are attributable to the action of the coagulants rather than to natural settling or degradation processes occurring in the raw water.

Raw water samples were collected from the Río Grande de Añasco at the intake point of the municipal water treatment plant (MWTP) and transferred into 1000 mL jars. Chitosan solutions, prepared in different acidic media, and sodium hypochlorite (NaOCl) were added at varying doses according to the turbidity level of each sample, as shown in Table 5.

Multiple jar tests were performed using each chitosan solution prepared in acid to evaluate turbidity removal performance. The dosage that resulted in the lowest turbidity was identified from each set of jar tests. Based on these preliminary results, a final jar test was conducted using doses close to the optimal value previously found. The dose that achieved the lowest turbidity in this final round was selected for comparative analysis of the total organic carbon (TOC) and TOC removal efficiency.

**Figure 7:**
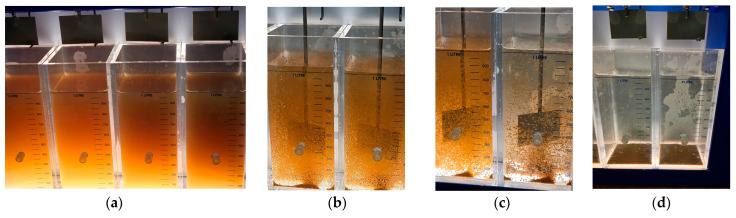
Visual representation of the jar test procedure applied to raw river water: (**a**) raw water before treatment; (**b**) coagulation under rapid mixing; (**c**) flocculation under slow mixing; (**d**) sedimentation phase showing floc settling.

To provide a visual reference for the treatment sequence, Figure 7 presents the key stages of the jar test process applied to raw river water. These images illustrate the initial appearance of untreated water (a), rapid mixing during the coagulation phase (b), slow mixing during flocculation (c), and the sedimentation phase (d). This sequence reflects the standard coagulation–flocculation protocol applied throughout the study and helps contextualize the subsequent turbidity and TOC removal results.

To assess the performance of the flocculation product, turbidity readings were taken at the inlet (NTU RW) and outlet (NTU out) of the flocculation unit at 32 min intervals (MWTP flocculation process), corresponding to the typical duration (30 min) of a complete flocculation cycle, as described by Letterman [5]. These measurements were used to calculate flocculation efficiency under varying raw water conditions. The efficiency of the flocculation process (EFP) was calculated using Equation (4). The graphical results revealed that among the tested doses, one specific dose of acetic acid consistently achieved both the lowest TOC concentration and the lowest turbidity level at the end of the treatment period. This outcome indicates that the selected dose was effective not only in removing dissolved organic matter but also in reducing particulate matter, making it the most efficient. These combined results are critical for identifying the optimal coagulant dose because both TOC and turbidity are key indicators of water quality. Accordingly, this dose was selected as the optimal dose and used as the reference condition in the comparative analysis of TOC removal efficiencies among the different acids. Selecting the dose that delivers the best combined performance ensures consistency and reliability when evaluating the effectiveness of coagulants across treatments. The jar test procedure was designed to replicate the actual coagulation–flocculation conditions applied at a municipal water treatment plant (MWTP). The tests followed a structured mixing protocol, as detailed in the Materials and Methods section.

#### 3.4.2. Chitosan in Acetic Acid Solution at Different Raw Water Turbidity Ranges

Turbidity range (300–600 NTU)

For raw water with an initial turbidity of 403 NTU, Table 6 presents the first stage of jar testing using chitosan dissolved in acetic acid, with a concentration range from 0.50 to 20.0 mg/L (Jar 5 representing the optimal dose). The second stage of the test, analyzed at 32 min of processing, is also shown in Table 6, with Jar 3 corresponding to the optimal concentration at that point.

The optimal dose was 8.00 mg/L, which resulted in a final turbidity of 6.40 NTU at 193 min, corresponding to Jar 3. As described previously, EFP was calculated using the inlet and outlet turbidity values to assess flocculation performance in this turbidity range. In this case, the raw water turbidity was 403 NTU and the outlet turbidity after flocculation with the selected dose was 9.18 NTU, resulting in an EFP of 97.7%. These findings indicate that chitosan effectively promoted the formation of dense and well-structured flocs by the end of the flocculation process, contributing to the high removal efficiency observed, and confirming the effectiveness of the selected dose under these raw water conditions.

The TOC concentration corresponding to Jar 3, with 8.00 mg/L of chitosan in acetic acid, was 2.76, 1.99, 1.99 to 32, 178, and 193 min, respectively, and the DOC concentration was 1.72 mg/L at 193 min. These results indicate a significant reduction in organic carbon content as flocculation progressed, suggesting that chitosan promoted the formation of dense, settleable flocs with a high binding capacity for organic matter.

Turbidity range (1000–3000 NTU)

For raw water with an initial turbidity of 1220 NTU, the first stage of jar testing using chitosan in acetic acid (concentration range: 3.00 to 30.0 mg/L) is presented in Table 7, with Jar 3 corresponding to the optimal concentration. The second stage focused on a refined dose range (8.00 to 14.0 mg/L) to determine the precise optimal dosage. Table 7 also includes the results from this second stage, with turbidity measurements taken at 32 min of processing. The optimal dose was found to be 10.0 mg/L, which achieved a turbidity of 12.7 NTU at 32 min and a final turbidity of 6.20 NTU at 193 min, corresponding to Jar 3.

As described previously, EFP was calculated using the inlet and outlet turbidity values to assess flocculation performance in this turbidity range. In this case, the raw water turbidity was 1220 NTU, and the outlet turbidity after flocculation with the selected dose was 12.7 NTU, resulting in an EFP of 99.0%. These results demonstrate a high level of removal by the end of the flocculation process, indicating that chitosan exhibited strong performance in floc formation and floc weight development, and confirmed the effectiveness of the selected dose under these raw water conditions.

The TOC concentration corresponding to Jar 3, with 10.0 mg/L of chitosan in acetic acid was 2.01, 2.02, and 1.98 at 32, 178, and 193 min, respectively, and the DOC concentration was 1.16 mg/L at 193 min. These results indicate a significant reduction in organic carbon content as flocculation progressed, suggesting that chitosan promoted the formation of dense, settleable flocs with a high binding capacity for organic matter.

Turbidity range (3000–6000 NTU)

For raw water with an initial turbidity of 5038 NTU, the first stage of jar testing evaluated a broad dose range (10.0–50.0 mg/L) to identify the optimal concentration. Table 8 presents the results from this first stage, with turbidity measurements taken at 32 min of processing. The second stage focused on a narrower dose range (16.0–23.0 mg/L) to refine the optimal dosage. Table 8 also includes the results from this second stage, with turbidity again analyzed at 32 min. The optimal dose was determined to be 17.0 mg/L, which resulted in a turbidity of 12.7 NTU at 32 min and a final turbidity of 4.68 NTU at 193 min, corresponding to Jar 2.

As described previously, EFP was calculated using the inlet and outlet turbidity values to assess flocculation performance in this turbidity range. In this case, the raw water turbidity was 5038 NTU and the outlet turbidity after flocculation with the selected dose was 12.2 NTU, resulting in an EFP of 99.8%. The observed removal efficiency at the end of the flocculation process suggests that chitosan was highly effective in promoting the aggregation and settling of suspended solids, leading to the formation of stable, high-density flocs, and confirming the effectiveness of the selected dose under these raw water conditions.

The TOC concentration corresponding to Jar 2, with 17.00 mg/L of chitosan in acetic acid was 2.50, 2.25, and 2.24 at 32, 178, and 193 min, respectively, and the DOC concentration was 1.27 mg/L at 193 min. These results indicate a significant reduction in organic carbon content as flocculation progressed, suggesting that chitosan promoted the formation of dense, settleable flocs with a high binding capacity for organic matter.

The experimental percentage of TOC removal (% TOC removal exp) was calculated from the data obtained using Equation (5) and compared with the theoretical value estimated using Equation (2). As shown in Table 9, the TOC removal performance and flocculation efficiency were evaluated at different turbidity ranges using acetic acid. The results demonstrated that the efficiency of the coagulation–flocculation process varied with turbidity levels, with higher turbidity samples generally requiring higher doses of acetic acid and achieving greater TOC removal. This table presents the experimental TOC removal percentages and flocculation efficiencies at specific time points for each turbidity level. The coagulation–flocculation test results showed TOC removal that was consistent with the requirements set by the EPA and predictions based on the SUVA values. For samples with an alkalinity of 80 mg/L, the EPA required a 25% removal of TOC levels between 2 and 4 mg/L, and 35% removal for TOC levels above 4 mg/L. For the sample with 403 NTU (initial TOC of 3.67 mg/L), 35.2% removal was achieved, exceeding the minimum requirement of 25%. The sample with 1220 NTU (initial TOC of 4.85 mg/L) showed 59.0% removal, surpassing the required removal of 35%. For the sample with 5038 NTU (initial TOC of 8.00 mg/L), the removal reached 72.0%, which is well above the minimum requirement.

Regarding the predictions based on SUVA values, for the samples with turbidity of 403 NTU and 1220 NTU, the expected TOC removal was in the 40–60% range, whereas for the 5038 NTU sample, the prediction was 60–80%. The experimental results were 35.2%, 59.2%, and 72.0%, respectively, and were aligned with expectations based on the SUVA values.

Additionally, flocculation efficiency was high across all samples, with values of 97.7% for the 403 NTU sample, 99.0% for the 1220 NTU sample, and 99.8% for the 5038 NTU sample, indicating effective removal of suspended particles, and contributing to significant reductions in turbidity and TOC.

As shown in Table 9, the TOC removal performance and flocculation process efficiency were evaluated at different turbidity ranges using acetic acid, and the percentage removal of UV_254_ absorbance and DOC was calculated using the same formula described in Equation (5), based on the values measured at 193 min. These complementary parameters help to further characterize the nature and extent of organic matter removal. Specifically, for the 403 NTU sample, a 40.9% reduction in UV_254_ absorbance and 44.0% DOC removal was achieved. The reduction in UV_254_ indicates a decrease in the concentration of aromatic organic compounds, which are typically associated with humic substances and known precursors of disinfection byproducts (DBPs). DOC removal confirmed that a substantial portion of the dissolved organic matter was successfully removed through coagulation–flocculation.

For the 1220 NTU sample, 61.8% UV_254_ removal and 69.2% DOC removal were observed, indicating a higher removal efficiency, which is consistent with the increased initial organic load and dose applied. In the 5038 NTU sample, which had the highest turbidity levels tested, the UV_254_ and DOC removal rates were 76.2% and 78.5%, respectively. These high removal rates reflect the predominance of humic high-molecular-weight organic compounds in this sample, which are more amenable to removal via enhanced coagulation. Overall, these findings suggest that the application of chitosan in acetic acid was particularly effective in targeting both aromatic and hydrophilic fractions of natural organic matter (NOM), with higher turbidity waters yielding greater removal efficiencies.

The THM formation potentials (THMFP) before and after treatment are summarized in Table 3 and Table 9. A clear reduction in THMFP was observed across all turbidity levels, demonstrating the effectiveness of the coagulation–flocculation process using chitosan in acetic acid in minimizing the precursors associated with disinfection byproduct (DBP) formation.

For the 403 NTU sample, the THMFP in the raw water was 222 µg/L, which decreased to 103 µg/L after the treatment, a reduction of approximately 53.6%. Similarly, the 1220 NTU sample decreased from 314 µg/L to 120 µg/L (61.8% reduction), and the sample with the highest turbidity (5038 NTU) decreased from 587 µg/L to 177 µg/L (69.8% reduction). These substantial reductions in THMFP correlated with the removal of both TOC and DOC, which are key precursors in DBP formation.

The treatment achieved TOC removals of 35.2, 59.2, and 72.0% for the 403, 1220, and 5038 NTU samples, respectively. Likewise, DOC concentrations were significantly reduced, particularly in the high-turbidity samples, where humic and hydrophobic substances dominated and were more readily removed through enhanced coagulation.

The consistent presence of residual chlorine in the finished water (ranging from 0.52 to 0.57 mg/L) confirms that sufficient disinfectant was available to react with any remaining organic matter, yet the THMFP remained substantially lower than in the raw water. This suggests that the reduction in DBP precursors was the main factor driving the observed decrease in THMFP, rather than the variation in chlorine dosage.

**Figure 8:**
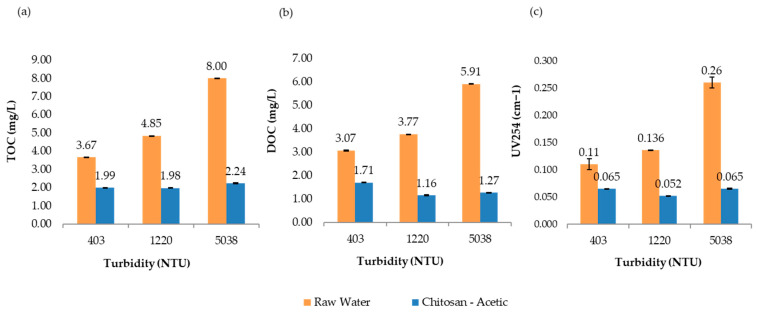
Jar test results for raw water and samples treated with chitosan dissolved in acetic acid solution. (**a**) Total organic carbon (TOC), (**b**) dissolved organic carbon (DOC), and (**c**) UV254 absorbance, evaluated across different turbidity ranges.

Figure 8 presents the comparative results of UV_254_ absorbance, total organic carbon (TOC), and dissolved organic carbon (DOC) measured at 193 min for all turbidity levels evaluated in the jar test process. Overall, the treatment not only met but exceeded the U.S. EPA requirements for TOC removal, effectively reducing the potential for trihalomethane (THM) formation in the treated water. These findings highlight the effectiveness and sustainability of chitosan in acetic acid as a viable alternative coagulant for improving drinking water quality.

#### 3.4.3. Chitosan in Lactic Acid Solution at Different Raw Water Turbidity Ranges

Turbidity range (300–600 NTU)

For the test performed with raw water at 403 NTU, the first stage of jar test testing is presented in Table 10, using chitosan in lactic acid with a concentration range of 0.50 to 20.0 mg/L. The second stage of jar testing focused on the dose range of 3.00 to 12.0 mg/L to determine the optimal dosage. Table 10 shows the jar test at the second stage with the turbidity analyzed at 32 min of process.

The best performance was observed at 8.00 mg/L, which corresponded to Jar 3, reaching a final turbidity of 12.0 NTU after 193 min. To determine the treatment efficiency, flocculation performance (EFP) was computed using turbidity reduction. Given an initial turbidity of 403 NTU and a value of 21.8 NTU after flocculation, the resulting EFP was 94.6%, which confirmed that this dose was highly effective under these conditions.

The TOC values recorded for Jar 3 (8.00 mg/L chitosan in lactic acid) at 32, 178, and 193 min were [2.30, 2.25, 2.25] mg/L, while the DOC concentration at 193 min was 1.50 mg/L. These results indicate a significant reduction in organic carbon content as flocculation progressed, suggesting that chitosan promoted the formation of dense, settleable flocs with a high binding capacity for organic matter.

Turbidity range (1000–3000 NTU)

For raw water exhibiting an initial turbidity of 1220 NTU, the first stage of jar testing with chitosan dissolved in lactic acid is presented in Table 11, using a concentration range of 3.00 to 30.0 mg/L. The second stage focused on a narrower dose range (8.00–14.0 mg/L) to determine the optimal concentration. Table 11 also includes the results from this second stage, with turbidity measurements taken at 32 min of processing.

The dose of 12.00 mg/L (Jar 4) was identified as the most efficient, producing a final turbidity of 9.96 NTU at 193 min. The flocculation efficiency (EFP) was assessed based on the decrease in turbidity from the raw water to the post-flocculation stage. With an initial turbidity of 1220 NTU and a value of 22.0 NTU after flocculation, the EFP reached 98.2%, confirming the high effectiveness of this dosage.

The TOC and DOC measurements for Jar 4 (12.00 mg/L) showed consistent reductions over time. These results indicate a significant reduction in organic carbon content as flocculation progressed, suggesting that chitosan promoted the formation of dense, settleable flocs with a high binding capacity for organic matter. Total organic carbon concentrations at 32, 178, and 193 min were [2.19, 2.18, 2.12] mg/L, while the dissolved organic carbon value at 193 min was 1.92 mg/L, indicating that the chitosan in lactic acid combination performed well in removing both dissolved and particulate organic fractions via floc formation.

Turbidity range (3000–6000 NTU)

For highly turbid water with an initial turbidity of 5038 NTU, the first stage of jar testing focused on a dose range of 10.0 to 50.0 mg/L to determine the optimal concentration. Table 12 presents the results from this first stage, with turbidity measured at 32 min of processing. The second stage involved testing chitosan dissolved in lactic acid across a narrower dose range (16.0 to 23.0 mg/L) to refine the determination of the most effective treatment dosage. Table 12 also includes the results from this second stage, with turbidity again analyzed at 32 min.

The optimal performance was observed at a dose of 21.00 mg/L (Jar 4), which yielded a final turbidity of 3.14 NTU at 193 min. To assess process performance, flocculation efficiency (EFP) was calculated using the initial and post-treatment turbidity values. In this case, turbidity was reduced from 5038 NTU to 8.84 NTU, resulting in an EFP of 99.8%, confirming excellent removal capacity even under severe turbidity conditions.

TOC concentrations for Jar 4 were [2.86, 2.82, 2.72] mg/L at 32, 178, and 193 min, while the DOC value at 193 min was 2.11 mg/L. These results indicate a significant reduction in organic carbon content as flocculation progressed, suggesting that chitosan promoted the formation of dense, settleable flocs with a high binding capacity for organic matter and the formation of compact and settleable flocs capable of capturing both particulate and dissolved organic fractions.

As shown in Table 13, the TOC removal performance, flocculation process efficiency, and additional organic matter removal parameters were evaluated at different turbidity levels by using chitosan in lactic acid. The results demonstrated that the efficiency of the coagulation–flocculation process increased with turbidity, with higher turbidity waters requiring higher doses of chitosan and achieving greater removal of total organic carbon (TOC). Specifically, for the sample with 403 NTU, a final TOC concentration of 2.25 mg/L was achieved, corresponding to 38.7% removal. For the 1220 NTU sample, the final TOC was 2.12 mg/L (56.3% removal), and for the most turbid sample, 5038 NTU, TOC was reduced to 2.72 mg/L (66.0% removal). All these values exceeded those of the U.S. EPA enhanced coagulation requirements: 25% removal for TOC levels between 2 and 4 mg/L and 35% removal for TOC levels greater than 4 mg/L, assuming an alkalinity above 60 mg/L. This confirms that the treatment applied not only met but also surpassed the minimum regulatory targets across all conditions tested.

Additionally, the data revealed that the most significant TOC reduction occurred within the first 32 min of the process, after which the concentrations remained relatively stable or decreased only slightly. This trend indicates that chitosan acted most effectively during the coagulation and flocculation phases, where it facilitated the rapid aggregation and removal of organic matter. This performance is characteristic of chitosan’s high charge density and bridging capabilities, supporting its role as an efficient natural coagulant.

Further characterization of organic matter removal was carried out by calculating the dissolved organic carbon (DOC) and UV_254_ absorbance removal at 193 min. These parameters help to clarify the extent and nature of natural organic matter (NOM) removed. For the 403 NTU sample, 51.1% DOC removal and 49.1% reduction in UV_254_ were observed. For the 1220 NTU sample, the DOC and UV_254_ removal rates were 49.1% and 39.7%, respectively, whereas the 5038 NTU sample showed the highest removal: 64.3% DOC and 66.5% UV_254_. The substantial reduction in UV_254_ confirms the effective elimination of aromatic and humic substances, which are key precursors of disinfection byproducts (DBPs), whereas DOC removal reflects the reduction in hydrophilic organic fractions. Compared with the theoretical expectations based on the SUVA values, the experimental outcomes align well.

The 403 and 1220 NTU samples exhibited SUVA values in the 3.5–3.6 L·mg^−1^·m^−1^ range, suggesting intermediate aromaticity and expected TOC removals of 40–60%, while the 5038 NTU sample had a SUVA of 4.39, predicting 60–80% removal. The actual TOC removal values (38.7%, 56.3%, and 66.0%) were within or near the predicted ranges, confirming the suitability of the applied treatment. The flocculation efficiency was also high for all samples (94.6%, 98.2%, and 99.8%), further supporting the effectiveness of chitosan in particle removal under the tested conditions.

As presented in Table 13, the coagulation–flocculation treatment using chitosan dissolved in lactic acid was effective in reducing THM formation potential (THMFP) across all turbidity ranges. Compared to the THMFP values of the raw water, a significant reduction was observed for each treated sample. For the 403 NTU sample, the raw water THMFP was 222 µg/L, which was reduced to 102 µg/L after treatment, a reduction of approximately 54.1%. In the case of 1220 NTU, THMFP decreased from 314 µg/L to 111 µg/L, resulting in a 64.6% reduction. The most notable change occurred in the 5038 NTU sample, where the raw THMFP concentration was 587 µg/L and dropped dramatically to 102 µg/L in the treated water, which is equivalent to a reduction of 82.6%. Although the TOC and DOC removal percentages in this set were slightly lower than those obtained with acetic acid (TOC removal of 38.7%, 56.3%, and 66.0% for increasing turbidity levels), the THMFP was better controlled, particularly for the sample with the highest turbidity. This suggests that lactic acid-based treatment was more effective at targeting specific fractions of NOM that are highly reactive in chlorine disinfection, especially those responsible for THM formation.

In terms of UV_254_, which serves as a proxy for aromatic organics, the removal was moderate, with final absorbance values of 0.056, 0.082, and 0.087 cm^−1^ for the 403, 1220, and 5038 NTU samples, respectively. These values indicate a reasonable reduction in the DBP precursors, particularly aromatic compounds. The residual chlorine levels in all the samples remained relatively high (0.95–1.21 mg/L), which ensured that disinfection was not limited by chlorine availability. Therefore, the lower THMFP values achieved with lactic acid likely stem from the more effective removal or alteration of highly reactive NOM components, not from reduced chlorination.

**Figure 9:**
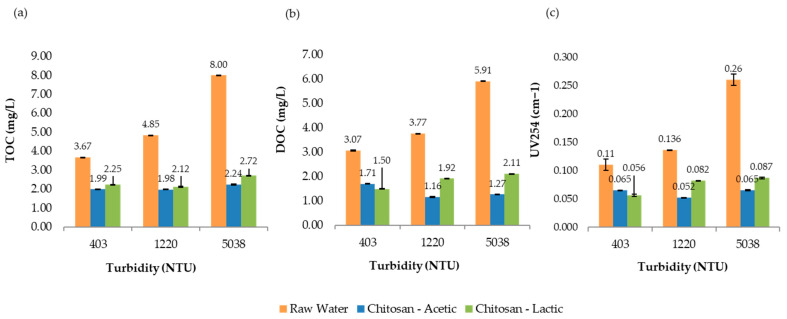
Jar test results for raw water and samples treated with chitosan dissolved in acetic and lactic acid solutions. (**a**) Total organic carbon (TOC), (**b**) dissolved organic carbon (DOC), and (**c**) UV254 absorbance, evaluated across different turbidity ranges.

Figure 9 presents the comparative results of UV_254_ absorbance, total organic carbon (TOC), and dissolved organic carbon (DOC) measured at 193 min for all turbidity levels evaluated in the jar test process. Overall, the results suggest that chitosan in lactic acid achieved strong THMFP control, especially at higher turbidity levels, despite achieving slightly lower TOC removal than the other acids. This highlights the importance of measuring both the organic matter concentration and reactivity when evaluating treatment effectiveness for DBP control.

#### 3.4.4. Chitosan in L-Ascorbic Acid Solution at Different Raw Water Turbidity Ranges

Turbidity range (300–600 NTU)

In tests using raw water with an initial turbidity of 403 NTU, the first stage of jar testing with chitosan dissolved in L-ascorbic acid is presented in Table 14, using a concentration range of 0.50 to 20.0 mg/L. A second, refined dose screening was conducted using concentrations between 2.00 and 12.0 mg/L to identify the most efficient coagulant dosage. Table 14 also presents the results from this second stage, with turbidity measurements taken at 32 min of processing.

The optimum result was obtained with an 8.00 mg/L dose, corresponding to Jar 3, where final turbidity was reduced to 6.47 NTU at 193 min. The flocculation efficiency (EFP), calculated from the reduction between initial and post-treatment turbidity, reached 95.0% (from 403 to 20.1 NTU), confirming the success of this treatment setup.

TOC analyses for Jar 3 at the three time points yielded concentrations of [2.35, 2.28, 2.21] mg/L, and the DOC concentration at 193 min was 1.87 mg/L. These data suggest that chitosan in L-ascorbic acid exhibited a strong capacity for organic matter removal, supporting the formation of well-structured flocs throughout the process.

Turbidity range (1000–3000 NTU)

The jar test was conducted using raw water with an initial turbidity of 1220 NTU. The first stage of testing with chitosan dissolved in acetic acid is presented in Table 15, using a concentration range of 3.00 to 30.0 mg/L. The second stage focused on a narrower dose range (6.00 to 15.0 mg/L) to determine the optimal coagulant dosage. Table 15 also includes the results from this second stage, with turbidity measurements taken at 32 min of processing.

Among the tested doses, 13.00 mg/L (Jar 4) delivered the best results, achieving a turbidity of 7.52 NTU at the final stage. The efficiency of the flocculation process was assessed by comparing inlet and outlet turbidity values. The reduction from 1220 NTU to 17.9 NTU yielded an EFP of 98.5%, confirming the suitability of this dose under high turbidity conditions.

For the same setup, the TOC and DOC levels significantly decreased throughout the treatment. Measured TOC concentrations at 32, 178, and 193 min were [2.28, 1.99, 1.98] mg/L, while the DOC concentration was 1.56 mg/L at 193 min. These findings suggest that this coagulant formulation supported the generation of compact flocs with a high affinity for both particulate and dissolved organic matter.

Turbidity range (3000–6000 NTU)

In the case of raw water with extremely high turbidity (5038 NTU), the first stage of jar testing focused on a dose range of 10.0 to 50.0 mg/L to identify the optimal concentration. Table 16 presents the results from this stage, with turbidity measured at 32 min of processing. The second stage involved testing chitosan dissolved in L-ascorbic acid across a narrower dose range (16.0 to 23.0 mg/L) to refine the determination of the most suitable treatment dosage. Table 16 also includes the results from this second stage, with turbidity again analyzed at 32 min.

Chitosan in L-ascorbic acid was tested within a dose range of 16.00–23.00 mg/L to identify the most effective coagulant concentration. The dose of 21.00 mg/L (Jar 4) achieved the best performance, reaching a final turbidity of 2.51 NTU at the end of the process. Turbidity dropped from 5038 NTU to 4.84 NTU after flocculation, yielding a flocculation efficiency (EFP) of nearly 100%. These results validated the effectiveness of the dose in handling water with extremely high turbidity levels. The removal of organic carbon was also significant. At 32, 178, and 193 min, TOC values were [2.16, 2.12, 2.06] mg/L, while the DOC level at 193 min was 1.39 mg/L. This suggests that chitosan in L-ascorbic acid supported effective floc formation and consistent removal of both particulate and dissolved organics through the various treatment stages.

As presented in Table 17, the TOC removal performance and flocculation efficiency were evaluated using chitosan in L-ascorbic acid at the three turbidity levels. The results showed a clear trend in which increased turbidity correlated with higher coagulant doses and enhanced organic matter removal. Most of the TOC reduction occurred within the first 32 min of treatment, suggesting that the chitosan–L-ascorbic acid formulation is particularly effective during the rapid coagulation–flocculation phase.

For the 403 NTU sample, an optimized dose of 8.00 mg/L resulted in a final TOC concentration of 2.21 mg/L, corresponding to a 39.8% TOC removal, which exceeds the 25% EPA minimum requirement for initial TOC levels between 2 and 4 mg/L. The DOC at the end of the process was 1.87 mg/L, indicating a 39.1% DOC reduction, and the UV_4254_ absorbance dropped by 39.1% (0.067 cm^−1^ at 193 min), highlighting effective removal of aromatic compounds. These values suggest moderate removal of hydrophobic fractions, consistent with the expectations for raw water with intermediate SUVA values.

For the 1220 NTU sample, a dose of 13.0 mg/L produced a final TOC of 1.98 mg/L from an estimated initial value of 4.85 mg/L, equating to a 59.2% TOC removal, again exceeding the 35% EPA requirement. The DOC decreased to 1.56 mg/L, representing a 58.6% DOC reduction, and the UV absorbance declined by 51.5%, suggesting the effective elimination of aromatic, humic-type organics. These outcomes align well with the theoretical SUVA-based TOC removal predictions in the 40–60% range for waters with intermediate aromatic contents. In the most turbid sample (5038 NTU), treated with 21.0 mg/L of chitosan–lactic acid, TOC was reduced to 2.06 mg/L, achieving a 74.3% removal, which is well above the 35% EPA target. The DOC was reduced to 1.39 mg/L (76.4% DOC removal), and UV_4254_ absorbance dropped by 70.8%, confirming a strong reduction in high-molecular-weight, aromatic organic matter typical of highly turbid river water dominated by humic substances. This performance is consistent with the theoretical SUVA removal range of 60–80% for waters with a high aromatic content. Across all turbidity levels, flocculation efficiency remained high, ranging from 95.0% to 99.9%, indicating excellent particle aggregation and sedimentation following the treatment. These findings reinforce the effectiveness of chitosan–L-ascorbic acid as a natural coagulant, particularly for water sources rich in humic substances and NOM with an aromatic character.

The effectiveness of chitosan dissolved in L-ascorbic acid in reducing trihalomethane formation potential (THMFP) was evaluated at three turbidity levels, as presented in Table 17. The comparison between the THMFP of raw water and that of finished water (FW) demonstrated a significant reduction across all conditions tested.

At 403 NTU, the raw water had a THMFP of 222 µg/L, while the finished water showed 177 µg/L, corresponding to a 20.3% reduction. For the 1220 NTU condition, the THMFP decreased more markedly from 314 µg/L in the raw water to 102 µg/L in the treated sample, a 67.5% reduction. At the highest turbidity level (5038 NTU), THMFP decreased from 587 µg/L to 108 µg/L, achieving an impressive 81.6% reduction. These results suggest that L-ascorbic acid was highly effective at minimizing DBP precursors, particularly in waters with higher turbidity.

**Figure 10:**
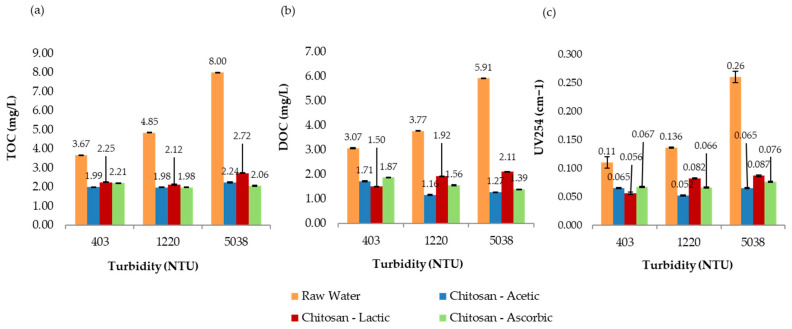
Jar test results for raw water and samples treated with chitosan dissolved in acetic, lactic, and L-ascorbic solutions. (**a**) Total organic carbon (TOC), (**b**) dissolved organic carbon (DOC), and (**c**) UV_254_ absorbance, evaluated across different turbidity ranges.

The final TOC removal percentages (39.8%, 59.2%, and 74.3%, respectively) aligned well with the observed THMFP reductions. For the most turbid sample, both TOC and DOC removal were the highest (TOC: 74.3%; DOC: 1.39 mg/L). The UV_254_ absorbance values (ranging from 0.066 to 0.076) also indicate notable removal of aromatic organics, which are closely linked to THM formation. Additionally, the relatively low residual chlorine levels across all samples (0.54–0.84 mg/L) suggest that chlorine demand was adequately managed post-treatment. Figure 10 presents the comparative results of UV_254_ absorbance, total organic carbon (TOC), and dissolved organic carbon (DOC) measured at 193 min for all turbidity levels evaluated in the jar test process. Overall, these findings highlight the potential of l-ascorbic acid as a promising co-solvent for chitosan in water treatment, particularly for effectively reducing DBP precursors in high-turbidity scenarios.

#### 3.4.5. Aluminum Chlorohydrate (GC 850) to Jar Test Analysis at Different Turbidity Ranges

The jar test concentrations for GC850 (aluminum chlorohydrate, ACH) were determined based on the dosage routinely applied at the municipal water treatment plant (MWTP) during the sampling period. As a result, no preliminary dose optimization tests were conducted for this coagulant, in contrast to the multi-stage jar testing approach employed for chitosan formulations.

Turbidity range (300–600 NTU)

In tests using raw water with 403 NTU turbidity, the jar test performance with GC850 (ACH) is presented in Table 18. Jar 4, which achieved the lowest turbidity value of 0.89 NTU, was selected as the optimal dose and analyzed for UV_254_ absorbance, total organic carbon (TOC), and dissolved organic carbon (DOC) at 193 min.

A coagulant dose of 12 mg/L, equivalent to the concentration applied at the municipal water treatment plant (MWTP) for that turbidity range, was used. In the full-scale process, the MWTP achieved a sedimentation turbidity of 3.22 NTU and a final filtered water turbidity of 0.23 NTU.

Turbidity range (1000–3000 NTU)

In tests using raw water with 1220 NTU turbidity with GC850 (ACH), the jar test performance is presented in Table 19. Jar 4, which achieved the lowest turbidity value of 2.64 NTU, was selected as the optimal dose and analyzed for UV_254_ absorbance, total organic carbon (TOC), and dissolved organic carbon (DOC) at 193 min. A coagulant dose of 16 mg/L, equivalent to the concentration applied at the municipal water treatment plant (MWTP) for that turbidity range, was used. In the full-scale process, the MWTP achieved a sedimentation turbidity of 4.01 NTU and a final filtered water turbidity of 0.18 NTU.

Turbidity range (3000–6000 NTU)

In tests using raw water with 5038 NTU turbidity with GC850 (ACH), the jar test performance is presented in Table 20.

Jar 3, which achieved the lowest turbidity value of 3.25 NTU, was selected as the optimal dose and analyzed for UV254 absorbance, total organic carbon (TOC), and dissolved organic carbon (DOC) at 193 min. A coagulant dose of 19.0 mg/L, equivalent to the concentration applied at the municipal water treatment plant (MWTP) for that turbidity range, was used. In the full-scale process, the MWTP achieved a sedimentation turbidity of 4.31 NTU and a final filtered water turbidity of 0.22 NTU.

As presented in Table 21, the TOC removal performance, flocculation efficiency, and disinfection byproduct precursor reduction were evaluated using GC850 (aluminum chlorohydrate) at three turbidity levels. Unlike the chitosan treatments, the dosage selection for GC850 was based on the operational dose applied at the municipal water treatment plant (MWTP) during the study period, without prior optimization through staged jar tests.

At 403 NTU, a dose of 12.0 mg/L reduced the TOC from 3.67 mg/L (raw water) to 2.01 mg/L at 193 min, achieving a 45.2% removal rate, which surpasses the U.S. EPA minimum requirement of 25% for water sources with initial TOC concentrations between 2 and 4 mg/L. The DOC concentration was lowered to 1.86 mg/L, and UV254 absorbance dropped significantly to 0.027 cm^−1^, indicating efficient removal of aromatic organic matter. The flocculation efficiency was 99.8%, and the THM formation potential (THMFP) decreased from 222 µg/L (raw water) to 105 µg/L, representing a 52.7% reduction. Residual chlorine measured 1.42 mg/L, suggesting an effective oxidation process with manageable chlorine demand.

For the 1220 NTU sample, 16.0 mg/L of GC850 reduced the TOC from 4.85 mg/L to 2.12 mg/L (43.7% removal), also exceeding the EPA target of 35% for this TOC range. DOC decreased to 1.91 mg/L, and UV254 absorbance reached a low of 0.015 cm^−1^. The THMFP dropped from 314 µg/L to 112 µg/L (64.3% reduction), and flocculation efficiency remained high at 99.8%. Residual chlorine was 0.79 mg/L.

**Figure 11:**
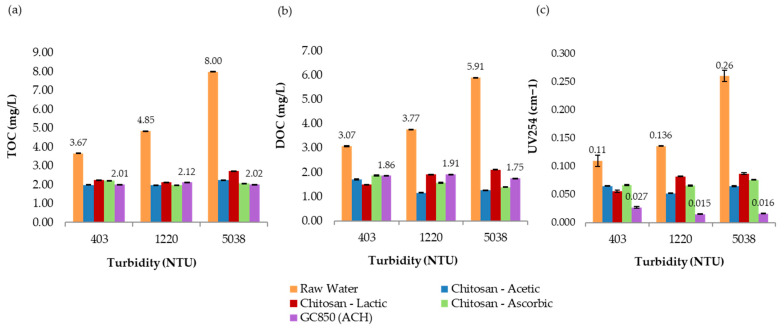
Jar test results for raw water and samples treated with GC850 (ACH) and chitosan dissolved in different acidic solutions. (**a**) Total organic carbon (TOC), (**b**) dissolved organic carbon (DOC), and (**c**) UV_254_ absorbance, evaluated across different turbidity ranges.

**Figure 12:**
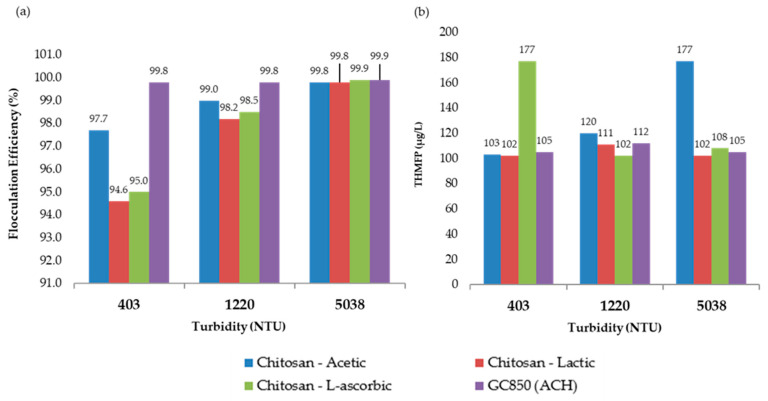
(**a**) Flocculation efficiency and (**b**) Trihalomethane formation potential (THMFP) for chitosan dissolved in different acidic solutions and the conventional coagulant ACH, evaluated across all turbidity ranges.

**Figure 13:**
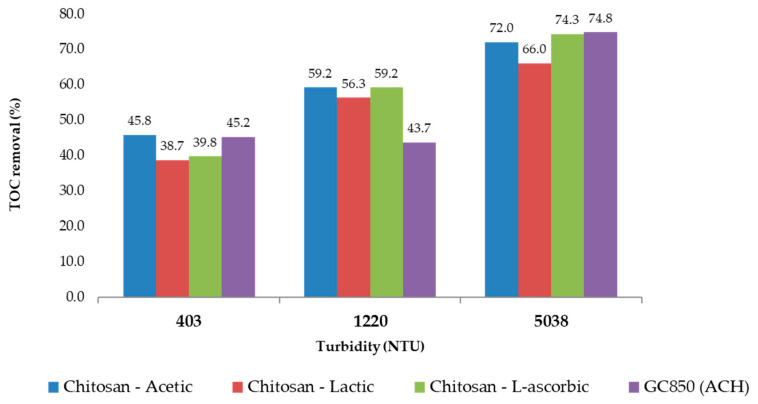
Total organic carbon (TOC) removal during jar testing using chitosan dissolved in different acidic solutions and the conventional coagulant ACH, evaluated across all turbidity ranges.

In the most turbid sample (5038 NTU), 19.0 mg/L of GC850 reduced TOC from 8.00 mg/L to 2.02 mg/L, achieving a 74.8% removal—well above the 35% EPA minimum. DOC was reduced to 1.75 mg/L, and UV254 absorbance dropped to 0.016 cm^−1^. These reductions reflect effective removal of humic, hydrophobic NOM fractions commonly associated with high SUVA values and turbidity. THMFP was reduced from 587 µg/L (raw water) to 105 µg/L (82.1% reduction), confirming significant removal of DBP precursors. Residual chlorine remained within a controlled range at 1.86 mg/L, with flocculation efficiency reaching 99.9%. Figure 11 presents the comparative results of UV_254_ absorbance, total organic carbon (TOC), and dissolved organic carbon (DOC) measured at 193 min for all turbidity levels evaluated in the jar test process. Figure 12 presents a comparison between chitosan and ACH regarding both flocculation efficiency and THMFP across all tested turbidity levels. Figure 13 shows total organic carbon (TOC) removal during jar testing using chitosan dissolved in different acidic solutions and the conventional coagulant ACH, evaluated across all turbidity ranges. Overall, GC850 demonstrated strong coagulation performance and substantial removal of TOC and THM precursors, particularly under high-turbidity conditions. However, the relatively high residual chlorine levels observed may warrant further consideration for post-treatment optimization.

### 3.5. Comparative Analysis of Chitosan Acidic Solutions and Conventional Coagulant Performance, Including the Literature

Each acid used (acetic, lactic, and L-ascorbic) affects the solubility, reactivity, and stability of chitosan in solution differently. Figure 11 and Figure 12 present a consolidated comparison of the performance of chitosan dissolved in three organic acids (acetic, lactic, and L-ascorbic) versus the conventional coagulant GC850 (ACH), across three turbidity levels (403, 1220, and 5038 NTU). As shown in Figure 13, TOC removal improved with increasing turbidity for all treatments. Chitosan in L-ascorbic acid achieved the highest removal at high turbidity (74.3% at 5038 NTU), closely matching GC850 (74.8%). At low turbidity (403 NTU), chitosan in acetic acid showed removal rates closest to GC850. All chitosan-based treatments met or exceeded the EPA’s minimum TOC removal requirement (25%) for low-alkalinity waters, aligning with theoretical expectations based on SUVA values.

Final TOC values at 193 min (Table 17) confirmed that chitosan in L-ascorbic acid and GC850 (Table 21) achieved the lowest residual TOC across all turbidity conditions, indicating effective organic matter reduction. Chitosan in acetic acid also performed well, particularly at moderate to high turbidity.

Final UV_254_ absorbance values (Figure 11) indicate significant reductions in aromatic and hydrophobic compounds. GC850 achieved the lowest UV_254_ values, followed closely by chitosan in acetic and L-ascorbic acids, especially at high turbidity. This suggests effective removal of aromatic natural organic matter, which is crucial for limiting disinfection byproduct (DBP) formation.

DOC removal varied by acid type and turbidity level. Chitosan in acetic acid performed best at medium turbidity, while GC850 showed consistent results across all conditions. At high turbidity, L-ascorbic acid matched the DOC reduction of GC850. At 5038 NTU, acetic acid achieved the highest DOC (78.5%) and UV_254_ (76.2%) removal, likely due to excellent dispersion and exposure of active sites. Its low viscosity enhances polymer mobility, promoting stable and cohesive floc formation [23,24]. Additionally, its simple carboxylic structure facilitates effective protonation of chitosan amino groups, improving solubility. In contrast, lactic acid exhibited higher viscosity, which may hinder chitosan dispersion and reduce charge neutralization efficiency [25]. Despite this, it achieved acceptable removals (TOC: 66%, UV_254_: 66.5%), potentially supported by its α-hydroxy acid structure, which fosters intermolecular interactions that increase viscosity and affect flocculation dynamics.

L-ascorbic acid, a strong and reducing acid, demonstrated high TOC (74.3%) and THMFP (81.6%) removal despite slightly lower DOC performance. This may be due to its ability to interact with aromatic precursors and modify the structure of some organics, making them more accessible for coagulation. Its multiple hydroxyl groups may also influence chitosan solubility and solution stability [26].

Figure 12b shows that THMFP was lowest for GC850 and chitosan in lactic acid, indicating effective DBP precursor removal. While L-ascorbic acid yielded high TOC removal, its higher THMFP may result from residual organics remaining reactive with chlorine. Figure 12a confirms that all treatments achieved flocculation efficiencies above 94%. At high turbidity, both chitosan in L-ascorbic acid and GC850 reached or exceeded 99.9%, demonstrating that properly formulated chitosan can rival conventional coagulants in clarification processes.

The observed variation in TOC, DOC, and UV_254_ removal across turbidity levels can be attributed, in part, to differences in the nature and concentration of natural organic matter (NOM) present in the raw water. At lower turbidity levels (403 and 1220 NTU), SUVA values (~3.6 L·mg^−1^·m^−1^) suggest intermediate aromaticity and a mixture of humic and non-humic substances, including hydrophilic components. This type of NOM is typically more challenging to remove, especially the non-humic, low-molecular-weight fractions, which are less prone to coagulation. As a result, TOC and DOC removal at these turbidity levels was moderate, and performance differences between acids became more evident due to their impact on chitosan’s solubility and interaction with specific NOM fractions. In contrast, at 5038 NTU, the NOM profile shifted notably. A higher SUVA value of 4.39 L·mg^−1^·m^−1^ indicates dominance of aromatic, humic, and hydrophobic compounds, which are more amenable to removal by coagulation and flocculation processes due to their larger molecular size and higher reactivity. This aligns with the observed increase in TOC and UV_254_ removal across all treatments at this turbidity level. Both GC850 and chitosan in L-ascorbic acid achieved TOC removals exceeding 74%, while UV_254_ absorbance was also significantly reduced, reflecting effective removal of aromatic DBP precursors.

This behavior is consistent with theoretical TOC removal estimates and EPA guidelines, further validating the applicability of chitosan in treating waters with varying organic loads. Previous studies have addressed different aspects of chitosan’s performance. Ampai Soros et al. concluded that chitosan has the potential to serve as an effective alternative coagulant for turbidity removal in water, demonstrating its applicability under moderate turbidity conditions [27].

Tomoko Takaara investigated the use chitosan as a coagulant aid. The study found that chitosan could replace polyacrylamide, contributing to improved treated water safety by reducing potential health risks [10].

R. Fabris et al. reported that chitosan was highly effective for particle removal at doses significantly lower than those required by conventional inorganic coagulants. However, chitosan alone was not particularly effective for dissolved organic carbon (DOC) removal, especially when used as the sole treatment step. When applied as the final clarification stage of a multi-step process, it showed limited turbidity reduction, likely due to specific flocculation requirements [28].

Furthermore, Eleanor B. Holmes et al. evaluated chitosan as a coagulation–flocculation pretreatment to enhance the performance of intermittently operated slow sand filtration systems. They reported reductions in bacteria, viruses, and turbidity when water was pre-treated with 10 mg/L of chitosan followed by flocculation [29].

Building upon this prior work, the present study demonstrates the effectiveness of chitosan as a primary coagulant under significantly higher turbidity conditions (up to 5038 NTU), while also evaluating the removal of TOC, DOC, and UV_254_. This provides a broader and more comprehensive assessment of chitosan’s performance, especially in challenging raw water matrices, and highlights its potential as a sustainable coagulant for improving treated water quality and reducing health risks.

Overall, the results support the use of chitosan—especially when formulated with L-ascorbic or acetic acid—as a viable natural coagulant, capable of matching or approaching the performance of conventional coagulants across multiple treatment metrics. UV_254_ reduction indicates the effective removal of aromatic DBP precursors, reinforcing the environmental relevance of these treatments.

### 3.6. Translating Laboratory Results to Real-World Applications in Water Treatment

#### 3.6.1. Limitations of Jar Tests and Potential for Real-Scale Implementation

Jar test experiments, while essential for optimizing coagulant doses and understanding coagulation–flocculation mechanisms under controlled conditions, present certain limitations. These include the use of fixed mixing speeds, ideal settling conditions, and small volumes that do not reflect the hydraulic dynamics, flow variations, and chemical interferences present in full-scale treatment plants. Consequently, performance metrics such as TOC or DOC removal may vary when scaled up, and operational challenges such as sludge handling or coagulant mixing efficiency cannot be fully assessed through bench-scale testing alone. Nevertheless, despite these known limitations, jar testing remains an approved and widely accepted method by regulatory agencies for process control in water treatment facilities, as it provides a reliable indication of how a specific coagulant dose may perform under actual plant conditions.

To assess the practical value of chitosan as a coagulant, real-scale or pilot-scale studies are necessary. These can explore two key application strategies: (1) using chitosan as a primary coagulant, especially in high-turbidity, NOM-rich waters, and (2) applying chitosan as a coagulant aid alongside traditional agents such as aluminum sulfate or ferric chloride. The latter approach has shown promise in reducing required doses of conventional chemicals, improving floc formation, and lowering chlorine demand, thereby helping meet DBP regulations. Real-scale evaluations would also allow for assessing sludge characteristics, operational costs, and long-term sustainability.

#### 3.6.2. Factors Influencing Chitosan Coagulation Performance in Water Treatment

The results obtained in this study demonstrate that the efficiency of chitosan as a natural coagulant varies significantly depending on the acid solvent used and the turbidity level of the raw water. This variation can be explained by a combination of physicochemical factors related to the nature of chitosan, the composition of the natural organic matter (NOM) present in the water, and the influence of the acidic medium on the structure and charge of the polymer.

The chitosan used, with a degree of deacetylation of 75% and a molecular weight of 460 kDa, contains amino groups (-NH_2_) that, when protonated in an acidic medium, acquire a positive charge (-NH_3_^+^). This allows electrostatic interactions with anionic organic compounds, such as humic and fulvic acids, which are commonly found in NOM.

The SUVA values obtained for the raw water (>3.5 L/mg·m) indicate a predominance of humic substances with high aromaticity and hydrophobicity. These characteristics enhance removal by flocculation and adsorption onto cationic polymers. This chemical affinity becomes particularly significant under high-turbidity conditions, where the elevated concentration of suspended particles and aromatic compounds provides additional sites for interaction. The selection of acetic acid, lactic acid, and L-ascorbic acid as solvents for chitosan dissolution was driven by key considerations related to their chemical characteristics, environmental relevance, and practical suitability for drinking water applications. These organic acids are naturally occurring in aquatic and terrestrial ecosystems and are commonly produced through microbial or plant metabolic activity, which aligns with the sustainability goals of this study [30,31,32].

Additionally, their biodegradable nature minimizes environmental risks when compared to inorganic acids, such as hydrochloric or sulfuric acid, which may introduce corrosion or toxic byproducts into the treated water [33]. Importantly, these acids also provide the necessary protonation environment (pH ~3.5–4.5) for dissolving chitosan. Due to their pKa values, acetic acid (~4.76), lactic acid (~3.86), and L-ascorbic acid (~4.2) are suitable for efficient amino group protonation on the chitosan polymer chain [34,35,36]. Their history of safe use in the food and pharmaceutical industries further supports their feasibility for drinking water applications. Both acetic and lactic acid are classified as GRAS (Generally Recognized As Safe) by regulatory agencies, and L-ascorbic acid is an essential vitamin, which collectively reduces concerns about toxicity [37].

This combination of natural origin, low environmental impact, suitable chemical reactivity, and regulatory acceptance underscores the relevance of these acids as effective and sustainable solvents for chitosan-based water treatment systems.

The removal efficiency of TOC, UV_254_, and THMFP increased notably with turbidity levels. At 403 NTU, removal rates were modest (TOC 35–40%, UV_254_ < 50%, THMFP 20–53%), which is consistent with a lower concentration of organic matter and less reactive NOM. At 1220 NTU, a significant increase was observed across all parameters, highlighting the impact of higher colloidal load and greater aromatic content. At 5038 NTU, the best results were achieved: the high concentration of NOM, its humified nature (SUVA of 4.39), and greater contact potential between chitosan and THM precursors enabled removal efficiencies exceeding 70% for all indicators.

These results confirm that chitosan is particularly effective in scenarios where the water is heavily loaded with aromatic NOM and suspended particles. Under such conditions, coagulation is enhanced through charge neutralization, interparticle bridging, and selective adsorption mechanisms.

The choice of acid solvent has a significant impact on the performance of chitosan as a coagulant, modulating its colloidal behavior, adsorption capacity, and affinity for organic substances. Acetic acid appears to offer the best combination of efficiency and stability, closely followed by L-ascorbic acid. Turbidity acts as an enhancing factor in the coagulation process by increasing the availability of interaction sites and the amount of organic matter to be removed.

The practical application of chitosan as a coagulant in water treatment processes is supported not only by its demonstrated effectiveness in TOC, DOC, and UV_254_ removal at varying turbidity levels but also by its economic feasibility. Based on dosing ranges observed in this study (2.00–21.0 mg/L depending on turbidity), an estimated 2–3 kg of chitosan powder would be required per 1,000,000 liters of treated water. The bulk market price of technical-grade chitosan typically ranges from 10 to 20 USD/kg, resulting in a treatment cost of approximately USD 20–60 per million litters. When compared with traditional coagulants such as aluminum sulfate, which can cost upwards of USD 140 per million liters (at typical dosages), chitosan presents a competitive alternative [38].

Additionally, chitosan offers environmental and health benefits as a biodegradable, non-toxic polymer derived from renewable sources, mainly crustacean shells. While it is not yet standardized as a disinfectant by regulatory agencies like the EPA, its safety profile is well established, and its use in water treatment is supported by multiple studies. Challenges related to storage conditions and stability during large-scale processing remain areas for further research; however, the current evidence suggests chitosan’s strong potential as a sustainable and cost-effective coagulant in water purification systems.

#### 3.6.3. Recommendations for Water Treatment Facilities

Chitosan powder must be stored under specific environmental conditions to preserve its physicochemical integrity and coagulant efficiency. Temperature is a critical factor; chitosan should be kept in a cool environment, ideally between 2 °C and 8 °C, as exposure to temperatures above 40 °C may lead to moisture loss and polymer degradation, which negatively affects its mechanical strength and functional properties [39]. Additionally, because chitosan is a hygroscopic material, it readily absorbs moisture from the surrounding air. When relative humidity exceeds 60%, its water content can increase significantly, promoting swelling, plasticization, and hydrolytic degradation [39,40]. Therefore, proper packaging is essential. Chitosan should be stored in hermetically sealed, opaque containers made of materials that can protect it from light and moisture while maintaining stable internal conditions [41].

It is important to note that storage requirements may vary depending on the physical state and formulation of the chitosan product. While the conditions apply to the dry powder form, chitosan solutions or industrially prepared blends—particularly those formulated with specific acids or coagulant aids—may have distinct handling and preservation needs. In such cases, storage conditions should be aligned with the manufacturer’s specifications, including recommendations related to pH stability, temperature sensitivity, and shelf life. Ensuring proper storage tailored to the product’s formulation is essential to maintaining its coagulation performance and chemical stability over time.

Chitosan’s hygroscopic nature—its tendency to absorb moisture from the environment—can negatively impact its flowability and dosing accuracy. Improper storage conditions may lead to clumping, which hinders uniform application and reduces its performance as a coagulant [40]. Additionally, extended exposure to high humidity and elevated temperatures accelerates the hydrolysis of chitosan, resulting in a reduction of its molecular weight. This degradation compromises its structural integrity and diminishes its effectiveness in water treatment applications [39].

To ensure optimal performance and longevity of chitosan during storage and use, several best practices should be implemented. Inventory control is essential and should follow a first-in, first-out (FIFO) protocol to ensure that older stock is used first and within its effective shelf life. Environmental monitoring systems must be installed in storage areas to maintain appropriate humidity and temperature levels, thereby preventing premature degradation. It is also important to train operational personnel in proper handling and storage procedures to minimize the risk of contamination and physicochemical deterioration. Finally, periodic quality checks—such as assessing the physical appearance, flowability, and pH of chitosan in solution—should be routinely conducted to verify that the stored material remains suitable for use prior to application.

Meeting the growing regulatory pressures surrounding disinfection byproducts (DBPs), many of which are classified as potentially carcinogenic, remains a major challenge for water utilities. Although adopting biopolymer-based coagulants like chitosan may require additional investment in material procurement and storage infrastructure, these initial costs are offset by long-term benefits. These include reduced DBP formation, improved environmental sustainability, and enhanced protection of public health. From both municipal and private-sector perspectives, such benefits are not merely advantageous, but essential in developing resilient and future-oriented water treatment strategies.

## 4. Conclusions

This study demonstrated that chitosan, when solubilized in organic acids such as acetic, lactic, and L-ascorbic acid, is an effective natural coagulant capable of removing natural organic matter and reducing the formation potential of disinfection byproducts (DBPs) in drinking water treatment. Across varying turbidity levels, chitosan treatments achieved high flocculation efficiencies, with L-ascorbic and acetic acid formulations showing comparable performance to the conventional aluminum-based coagulant GC850.

The results support the viability of chitosan-based systems as sustainable alternatives to conventional coagulants. Their ability to reduce turbidity, TOC, DOC, UV_254_ absorbance, and THMFP under high NOM load conditions makes them particularly suitable for treating raw surface waters. These findings contribute to advancing natural coagulant applications and highlight the relevance of acid selection in optimizing chitosan performance for water treatment purposes.

## Figures and Tables

**Table 1:** Raw water sample parameters and calculated SUVA values.

Turbidity Range (NTU)	TOC RW (mg/L)	UV254 (cm^−1^)	DOC RW (mg/L)	pH/T (°C)	SUVA (L/mg·m)
403	3.67	0.110	3.07	7.40/25.9	3.58
1220	4.85	0.136	3.77	7.50/24.6	3.61
5038	8.00	0.260	5.91	7.52/24.1	4.39

Notes: UV_254_ = ultraviolet absorbance at 254 nm; DOC RW = dissolved organic carbon in raw water; TOC RW = total organic carbon in raw water; T = temperature in degrees Celsius; SUVA = specific ultraviolet absorbance.

**Table 2:** Comprehensive characterization of raw water from MWTP (Río Grande de Añasco): turbidity, TOC, alkalinity, SUVA, removal requirements, and NOM characteristics.

Turbidity (NTU)	TOC (mg/L)	Alkalinity (mg/L)	SUVA (L·mg^−1^·m^−1^)	Theoretical TOC Removal (%)	EPA Removal Requirement (%)	Expected Removal Range (%)	Dominant NOM
403	3.67	80	3.58	58.1	25	40–60	Intermediate aromaticity; mixed humic/non-humic
1220	4.85	80	3.61	58.8	25	40–60	Intermediate aromaticity; mixed humic/non-humic
5038	8.00	76	4.39	76.1	35	60–80	High aromatic content; dominated by humic, hydrophobic substances

**Table 3:** TOC removal and THMFP in relation to turbidity levels.

Turbidity Range RW (NTU)	Theoretical TOC Removal (%)	THM Formation Potential (µg/L)
403.0	58.1	222.0
1220.0	58.8	314.0
5038.0	76.1	587.0

**Table 4:** Acid solutions prepared for chitosan dissolution (Stock: 1000 mg/L).

Acid	Physical State	Molar Mass (g/mol)	Purity (%)	Mass (g)	Volume (µL)	pH	Chitosan (g)
Acetic	Liquid	60.05	99.7	–	100	3.81	1.0004
Lactic	Liquid	90.08	86.0	–	100	3.53	1.0003
L-ascorbic	Solid	176.12	99.0	0.1724	–	3.79	1.0001

Note: Purity values were reported as provided by the respective manufacturers. Mass measurements were obtained using an analytical balance with a precision of four decimal places (±0.0001 g).

**Table 5:** Chitosan dose range and NaOCl concentration applied per turbidity range.

Turbidity (NTU)	Chitosan Dose (mg/L)	NaOCl Dose (mg/L)
403	0.50–20.0	3.00
1220	3.00–30.0	3.00
5038	10.0–50.0	3.60

**Table 6:** Jar test (Stages 1 and 2) using chitosan in acetic acid: identification of optimal coagulant dose based on turbidity removal performance.

Jar	Chitosan in Acetic Stage 1 (mg/L)	Turbidity Stage 1 (NTU)	Chitosan in Acetic Stage 2 (mg/L)	Turbidity Stage 2 (NTU)
1	0.50	35.1	3.00	17.4
2	2.00	26.3	5.00	9.99
3	3.00	20.1	8.00	9.18
4	6.00	12.3	10.0	38.5
5	8.00	10.4	12.0	44.0
6	10.0	36.1	-	-
7	14.0	51.3	-	-
8	16.0	63.9	-	-
9	18.0	75.2	-	-
10	20.0	91.8	-	-

**Table 7:** Jar test (Stages 1 and 2) using chitosan in acetic acid: identification of optimal coagulant dose based on turbidity removal performance.

Jar	Chitosan in Acetic Stage 1 (mg/L)	Turbidity Stage 1 (NTU)	Chitosan in Acetic Stage 2 (mg/L)	Turbidity Stage 2 (NTU)
1	3.00	56.7	8.00	35.1
2	6.00	42.4	9.00	20.1
3	9.00	18.5	10.0	12.7
4	12.0	35.2	12.0	30.5
5	15.0	38.3	14.0	35.4
6	18.0	46.0	-	-
7	21.0	61.4	-	-
8	24.0	73.9	-	-
9	27.0	86.2	-	-
10	30.0	102	-	-

**Table 8:** Jar test (Stages 1 and 2) using chitosan in acetic acid: identification of optimal coagulant dose based on turbidity removal performance.

Jar	Chitosan in Acetic Stage 1 (mg/L)	Turbidity Stage 1 (NTU)	Chitosan in Acetic Stage 2 (mg/L)	Turbidity Stage 2 (NTU)
1	10.0	75.2	16.0	16.8
2	15.0	25.0	17.0	12.2
3	20.0	46.3	19.0	24.3
4	25.0	85.0	20.0	44.7
5	30.0	87.1	23.0	54.6
6	35.0	94.3	-	-
7	40.0	99.2	-	-
8	45.0	110	-	-
9	50.0	122	-	-

**Table 9:** TOC removal performance and flocculation process efficiency using acetic acid at different turbidity ranges.

Turbidity (NTU)	Optimized Dose (mg/L)	TOC 32 min (mg/L)	TOC 178 min (mg/L)	TOC 193 min (mg/L)	DOC 193 min (mg/L)	UV_254_ 193 min (mg/L)	TOC Removal (%)	Flocculation Efficiency (%)	THMFP FW (µg/L)	Residual Cl (mg/L)
403 NTU	8.00	2.71	1.99	1.99	1.71	0.065	35.2	97.7	103	0.52
1220 NTU	10.0	2.01	2.02	1.98	1.16	0.052	59.2	99.0	120	0.57
5038 NTU	17.0	2.50	2.25	2.24	1.27	0.065	72.0	99.8	177	0.54

**Table 10:** Jar test (Stages 1 and 2) using chitosan in lactic acid: identification of optimal coagulant dose based on turbidity removal performance.

Jar	Chitosan in Lactic Stage 1 (mg/L)	Turbidity Stage 1 (NTU)	Chitosan in Lactic Stage 2 (mg/L)	Turbidity Stage 2 (NTU)
1	0.50	35.1	3.00	52.2
2	2.00	26.3	6.00	49.1
3	3.00	56.4	8.00	21.8
4	6.00	51.2	10.0	33.2
5	8.00	21.8	12.0	45.8
6	10.0	36.0	-	-
7	14.0	46.5	-	-
8	16.0	55.1	-	-
9	18.0	62.1	-	-
10	20.0	78.4	-	-

**Table 11:** Jar test (Stages 1 and 2) using chitosan in lactic acid: identification of optimal coagulant dose based on turbidity removal performance.

Jar	Chitosan in Lactic Stage 1 (mg/L)	Turbidity Stage 1 (NTU)	Chitosan in Lactic Stage 2 (mg/L)	Turbidity Stage 2 (NTU)
1	3.00	166	8.00	166
2	6.00	136	9.00	136
3	9.00	77.5	10.0	77.5
4	12.0	23.1	12.0	22.0
5	16.0	45.4	14.0	40.2
6	18.0	58.2	-	-
7	22.0	66.8	-	-
8	24.0	89.6	-	-
9	26.0	103	-	-
10	30.0	115	-	-

**Table 12:** Jar test (Stages 1 and 2) using chitosan in lactic acid: identification of optimal coagulant dose based on turbidity removal performance.

Jar	Chitosan in Lactic Stage 1 (mg/L)	Turbidity Stage 1 (NTU)	Chitosan in Lactic Stage 2 (mg/L)	Turbidity Stage 2 (NTU)
1	10.0	85.3	16.0	12.5
2	15.0	22.4	17.0	11.3
3	20.0	11.8	20.0	10.4
4	25.0	19.2	21.0	8.84
5	30.0	35.1	23.0	15.2
6	35.0	41.1	-	-
7	40.0	48.9	-	-
8	45.0	62.4	-	-
9	50.0	75.0	-	-

**Table 13:** TOC removal performance using lactic acid at different turbidity ranges.

Turbidity (NTU)	Optimized Dose (mg/L)	TOC 32 min (mg/L)	TOC 178 min (mg/L)	TOC 193 min (mg/L)	DOC 193 min (mg/L)	UV_254_ 193 min (mg/L)	TOC Removal (%)	Flocculation Efficiency (%)	THMFP FW (µg/L)	Residual Cl (mg/L)
403 NTU	8.00	2.30	2.25	2.25	1.50	0.056	38.7	94.6	102	0.95
1220 NTU	12.0	2.19	2.18	2.12	1.92	0.082	56.3	98.2	111	1.21
5038 NTU	21.0	2.86	2.82	2.72	2.11	0.087	66.0	99.8	102	0.93

**Table 14:** Jar test (Stages 1 and 2) using chitosan in L-ascorbic acid: identification of optimal coagulant dose based on turbidity removal performance.

Jar	Chitosan in L-Ascorbic Stage 1 (mg/L)	Turbidity Stage 1 (NTU)	Chitosan in L-Ascorbic Stage 2 (mg/L)	Turbidity Stage 2 (NTU)
1	0.50	33.5	2.00	26.8
2	2.00	26.8	4.00	24.6
3	3.00	26.1	8.00	20.1
4	6.00	24.6	10.0	23.4
5	8.00	20.1	12.0	33.9
6	10.0	23.4	-	-
7	14.0	36.2	-	-
8	16.0	41.0	-	-
9	18.0	54.1	-	-
10	20.0	58.3	-	-

**Table 15:** Jar test (Stages 1 and 2) using chitosan in L-ascorbic acid: identification of optimal coagulant dose based on turbidity removal performance.

Jar	Chitosan in L-Ascorbic Stage 1 (mg/L)	Turbidity Stage 1 (NTU)	Chitosan in L-Ascorbic Stage 2 (mg/L)	Turbidity Stage 2 (NTU)
1	3.00	42.1	6.00	37.4
2	6.00	38.4	7.00	34.1
3	8.00	29.0	8.00	30.4
4	12.0	20.2	13.0	17.9
5	16.0	24.3	15.0	20.2
6	18.0	31.4	-	-
7	22.0	36.3	-	-
8	24.0	39.2	-	-
9	26.0	42.5	-	-
10	30.0	45.8	-	-

**Table 16:** Jar test (Stages 1 and 2) using chitosan in L-ascorbic acid: identification of optimal coagulant dose based on turbidity removal performance.

Jar	Chitosan in L-Ascorbic Stage 1 (mg/L)	Turbidity Stage 1 (NTU)	Chitosan in L-Ascorbic Stage 2 (mg/L)	Turbidity Stage 2 (NTU)
1	10.0	19.3	16.0	11.4
2	15.0	14.1	17.0	9.15
3	20.0	6.45	20.0	5.70
4	25.0	6.78	21.0	4.84
5	30.0	10.4	23.0	5.30
6	35.0	12.8	-	-
7	40.0	19.1	-	-
8	45.0	26.4	-	-
9	50.0	36.2	-	-

**Table 17:** TOC removal performance using L-ascorbic acid at different turbidity ranges.

Turbidity (NTU)	Optimized Dose (mg/L)	TOC 32 min (mg/L)	TOC 178 min (mg/L)	TOC 193 min (mg/L)	DOC 193 min (mg/L)	UV_254_ 193 min (mg/L)	TOC Removal (%)	Flocculation Efficiency (%)	THMFP FW (µg/L)	Residual Cl (mg/L)
403 NTU	8.00	2.35	2.28	2.21	1.87	0.067	39.8	95.0	177	0.84
1220 NTU	13.0	2.28	1.99	1.98	1.56	0.066	59.2	98.5	102	0.57
5038 NTU	21.0	2.16	2.12	2.06	1.39	0.076	74.3	99.9	108	0.54

**Table 18:** Jar test using GC850 (aluminum chlorohydrate, ACH); turbidity at 32 min of processing.

Jar	ACH (mg/L)	Turbidity (NTU)
1	8.00	2.18
2	10.0	1.92
3	11.0	1.52
4	12.0	0.89
5	14.0	2.10

**Table 19:** Jar test using GC850 (aluminum chlorohydrate, ACH); turbidity at 32 min of processing.

Jar	ACH (mg/L)	Turbidity (NTU)
1	12.0	5.10
2	14.0	4.80
3	15.0	4.26
4	16.0	2.64
5	17.0	3.81

**Table 20:** Jar test using GC850 (aluminum chlorohydrate, ACH); turbidity at 32 min of processing.

Jar	ACH (mg/L)	Turbidity (NTU)
1	16.0	12.2
2	18.0	10.3
3	19.0	3.25
4	20.0	4.16
5	21.0	4.39

**Table 21:** TOC removal performance using aluminum chlorohydrate at different turbidity ranges.

Turbidity (NTU)	Optimized Dose (mg/L)	TOC 32 min (mg/L)	TOC 178 min (mg/L)	TOC 193 min (mg/L)	DOC 193 min (mg/L)	UV_254_ 193 min (mg/L)	TOC Removal (%)	Flocculation Efficiency (%)	THMFP FW (µg/L)	Residual Cl (mg/L)
403 NTU	12.0	2.18	2.16	2.01	1.86	0.027	45.2	99.8	105	1.42
1220 NTU	16.0	2.30	2.21	2.12	1.91	0.015	43.7	99.8	112	0.79
5038 NTU	19.0	2.09	1.95	2.02	1.75	0.016	74.8	99.9	105	1.86

## Data Availability

The original contributions presented in the study are included in the article, further inquiries can be directed to the corresponding author.
